# Measuring Organizational Cultural Competence to Promote Diversity in Academic Healthcare Organizations

**DOI:** 10.1089/heq.2018.0007

**Published:** 2018-11-08

**Authors:** Jaya Aysola, Diana Harris, Hairong Huo, Charmaine S. Wright, Eve Higginbotham

**Affiliations:** ^1^ Division of General Internal Medicine, University of Pennsylvania, Philadelphia, Pennsylvania.; ^2^ Office of Inclusion and Diversity, Perelman School of Medicine, University of Pennsylvania, Philadelphia, Pennsylvania.; ^3^ Leonard Davis Institute of Health Economics, University of Pennsylvania, Philadelphia, Pennsylvania.; ^4^ Associate Designated Institutional Official for Health Equity and Inclusion, Graduate Medical Education, University of Pennsylvania, Philadelphia, Pennsylvania.

**Keywords:** diversity, healthcare disparities, inclusion, workforce

## Abstract

**Purpose:** To evaluate what drives respondent perceptions of health system organizational cultural competence.

**Methods:** We estimated associations between survey respondent (*n*=3506) demographic characteristics, length of employment, position, and place of work and their reported perceptions of institutional culture.

**Results:** In adjusted analyses, respondents self-identifying as non-Hispanic black versus non-Hispanic whites, females versus males, and lesbian/gay/bisexual/transgender/queer versus heterosexuals were significantly less likely to rank the cultural competence of their organization above average.

**Conclusion:** Minorities and women were less likely to rank their organization as culturally competent. Organizational efforts to achieve cultural competency would benefit from measuring this factor to target their efforts.

## Introduction

There is a growing call in medicine for education on how best to provide care to diverse populations in a culturally humble and effective way.^1,2^ A diverse workforce is essential to achieving this educational mission as well as delivering culturally effective and equitable care to diverse populations.^3,4^ Evidence suggests that successful organizations rely on blending diversity-related educational content with exposure to diverse individuals at the workplace.^5,6^ The rationale posited for why populations with equal access to care may experience unequal treatment is the presence of healthcare provider unconscious biases coupled with the institutional bias of healthcare systems, historically designed with a singular cultural perspective that inadvertently disadvantages some.^7^ A diverse workforce, which consists of individuals from varying cultures, faiths, backgrounds, and experiences, serves to combat institutional bias by bringing diverse minds with multiple cultural perspectives together to generate new medical knowledge and design healthcare delivery systems. Yet, how we promote inclusion, where all members of the workforce feel involved and empowered to thrive within healthcare organizations, remains an unanswered question.

Historically, diversity efforts within organizations have focused more on how to create a diverse applicant pool for recruitment over how to retain their existing talented and diverse workforce.^8,9^ This may be because of an insufficient understanding of how organizations can effectively operationalize inclusion. The Association of American Medical Colleges (AAMC) in collaboration with University of Massachusetts Medical School introduced the Diversity Engagement Survey (DES) to measure how effective organizations are at engaging a diverse workforce.^10,11^

We examined a subcomponent of this survey, organizational cultural competence factor (CCF), to better understand how our healthcare organizations can operationalize inclusion. Our primary objective was to evaluate associations between employee and student characteristics and perceptions of organizational cultural competence within a university-based healthcare system.

## Methods

We conducted a cross-sectional subanalysis of deidentified individual-level data from a 22-item DES. This survey was administered through email to medical, nursing, and dental students, trainees, faculty, and healthcare employees list-serves across our University-wide health system by DataStar^12^ with strict data privacy practices in efforts to preserve respondent anonymity.^13^ Three email reminders were sent and the survey was open for response from February to April of 2015. Survey completion was voluntary and not incentivized. Our institution was not part of the original 14 institutions that participated in the study to determine the construct and criterion validity of the DES instrument.^10^

The DES, which measures inclusiveness across three domains: (1) vision and purpose; (2) camaraderie; and (3) appreciation, was designed to reveal the aspects of institutional culture and social dynamics related to engagement and inclusion that have been shown to be strongly correlated with productivity and employee retention. We examined the associations between respondent characteristics and one component of this survey that measures organization cultural competence as our dependent variable. The University of Pennsylvania Institutional Review Board approved our study protocol.

### Independent variables

We examined several self-reported respondent characteristics including generational age group, gender, race/ethnicity, sexual orientation, belief system, disability status, primary language, position, length of employment, and organizational site (where a respondent spends the majority of his or her time working).

### Dependent variable

We focused on the CCF, which assesses perceptions of organizational culture and capacity to make creative use of its diverse workforce^10^ and includes the following four 5-point Likert scale questions to generate a score from 0 to 20:

1. In this institution, I have opportunities to work successfully in settings with diverse colleagues.
2. I believe my institution manages diversity effectively.
3. In my institution, I receive support for working with diverse groups and working in cross-cultural situations.
4. In this institution, there are opportunities for me to engage in service and community outreach.

### Statistical analysis

We examined associations between respondent characteristics and our primary outcome (CCF score) as both a continuous and binary variable (either above or below the mean score). We fitted multivariate linear and logistic regression models to estimate the associations of respondent characteristics with CCF score, adjusting for all other covariates, using generalized estimating equations with robust standard errors to account for clustering by site. We also ran our models including site as a covariate. To understand any differences by site, we determined the difference in adjusted rates (risk difference) by race/ethnicity and gender within sites and contrasted those risk differences to determine statistical significance. Two-tailed *p*-values and 95% confidence intervals (CIs) are reported for all covariates; *p*<0.05 was considered statistically significant.

## Results

From an available pool of 18,550 individuals, 19% or 3506 responded. The mean (standard deviation [SD]) of the CCF score was 15.4 (3.0) and the median (interquartile range [IQR]) was 16 (3), with 52.4% of the respondents (*n*=3506) characterizing cultural competence of their institution above the mean. Given a narrow IQR and no distinctions between our models estimating associations with CCF score as a continuous versus binary outcome, we chose to present our binary model results, for ease of interpretation. We found the Cronbach's α for the CCF four-item measure was 0.79 with significant correlation between the four survey questions. Table 1 shows the number and percentage of respondents by characteristic that ranked their organization's cultural competence as above versus below the mean. We found significant unadjusted associations between all examined covariates and ranking the CCF score above the mean.

**Table 1.** Comparisons of the Number and Percentage of Respondents by Characteristic That Ranked the Organizational Cultural Competence (Cultural Competence Factor Score) Above Versus Below Average

Respondent characteristic	Perceived organizational cultural competence (CCF score)		*p*
	Below mean	Above mean	
Generational age group, *n* (%)			0.002
1922–1944 (traditional)	24 (32.4)	50 (67.6)	
1945–1964 (baby boomers)	409 (45.0)	499 (55.0)	
1965–1980 (generation X)	523 (51.4)	495 (48.6)	
1981–2000 (millennials)	674 (46.7)	770 (53.3)	
Gender identity			<0.0001
Male	491 (41.1)	704 (58.9)	
Female	1145 (50.8)	1109 (49.2)	
Transgender/queer^a^	9 (56.3)	7 (43.8)	
Sex orientation			<0.0001
Heterosexual	1391 (45.8)	1646 (54.2)	
LGBTQ^b^	213 (60.0)	142 (40.0)	
Race/ethnicity			
Non-Hispanic white	1001 (44.2)	1263 (55.8)	
Non-Hispanic black	248 (67.6)	119 (32.4)	
Asian	151 (40.7)	220 (59.3)	
Hispanic	98 (45.2)	119 (54.8)	
Multi	60 (60.6)	39 (39.4)	
Other^c^	52 (56.5)	40 (43.5)	
Belief system			<0.0001
Christian	678 (43.9)	867 (56.1)	
Non-Christian^d^	774 (48.6)	819 (51.4)	
Decline to answer^e^	169 (59.3)	116 (40.7)	
Primary language			0.03
English	1533 (48.1)	1652 (51.9)	
Non-English	111 (41.1)	159 (58.9)	
Disability			0.005
Yes	43 (60.6)	28 (39.4)	
No	1504 (46.7)	1719 (53.3)	
Decline to answer^e^	90 (56.3)	70 (43.7)	
Length of time at institution			<0.0001
<1 year	225 (13.6)	324 (17.7)	
1–5 years	626 (37.8)	686 (37.5)	
5–10 years	297 (18.0)	243 (13.3)	
≥10 years	507 (30.6)	577 (31.5)	
Position			0.003
Executive	43 (32.8)	88 (67.2)	
Faculty/physician	398 (46.8)	453 (53.2)	
Housestaff/trainee/PhD student^f^	178 (43.9)	227 (56.1)	
Staff^g^	720 (50.0)	720 (50.0)	
Student^h^	297 (47.4)	330 (52.6)	
Other	9 (60.0)	6 (40.0)	
Area where majority of time is spent working			<0.0001
Children's hospital	164 (35.3)	301 (64.7)	
Dental school	61 (44.9)	75 (55.1)	
Health system affiliated hospitals^i^	417 (46.0)	490 (54.0)	
School of nursing	68 (58.1)	49 (41.9)	
School of social policy and practice	50 (55.6)	40 (44.4)	
School of medicine	609 (49.3)	626 (50.7)	
Other^j^	283 (53.4)	247 (46.6)	

^a^ Includes “transgender,” “other,” and “do not identify.”

^b^ Includes “lesbian/gay/homosexual/queer,” “bisexual,” and “other.”

^c^ Includes NA/AN, PI, and other (Reflects both unspecified and free text specified responses. Most common specified responses were Middle Eastern/Arab and South Asian/Asian Indian).

^d^ Includes all other categories (and Jewish).

^e^ Declined to answer includes both refused and/or missing responses.

^f^ Includes “resident/fellow/intern/postdoc and PhD student.”

^g^ Includes “staff” and “staff–manager level.”

^h^ Includes students affiliated with healthcare system (from schools of medicine, nursing, social policy and practice, and dentistry).

^i^ Includes the VA and all other adult care hospitals.

^j^ Includes outpatient practices, satellite sites, and/or services.

CCF, cultural competence factor; LGBTQ, lesbian/gay/bisexual/transgender/queer; NA/AN, Native American/American Native; PI, Pacific Islander; VA, Veterans Administration.

**FIG. 1.**
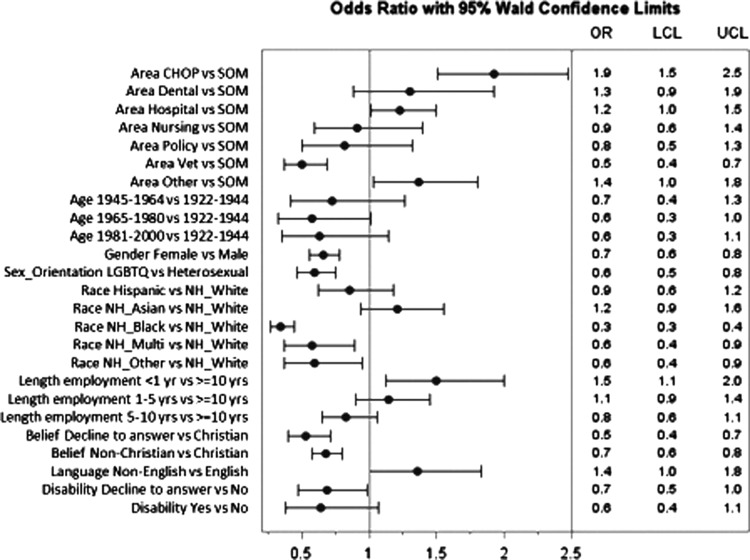
Adjusted ORs (95% confidence interval) of ranking organization CCF score above the mean. CCF, cultural competence factor; CHOP, Children's Hospital of Philadelphia; LCL, lower confidence level; LGBTQ, lesbian/gay/bisexual/transgender/queer; NH, non-Hispanic; OR, odds ratio; SOM, school of medicine; UCL, upper confidence level.

Figure 1 depicts the adjusted odds ratios (AORs) and 95% CIs of ranking CCF above the mean by self-identified respondent characteristics. Respondent characteristics significantly associated with CCF scores in adjusted models include gender identity, sexual orientation, race/ethnicity, site of work, length of employment, and belief system. Respondents were more likely to report low organizational cultural competence if they self-identified as non-Hispanic black as compared with non-Hispanic whites (AOR: 0.34; 95% CI: 0.28–0.44), females as compared with males (AOR: 0.66; 95% CI: 0.56–0.78), and lesbian/gay/bisexual/transgender/queer (LGBTQ) as compared with heterosexuals (AOR: 0.59; 95% CI: 0.46–0.76). In addition, those who identified as multiracial and “other” as compared with non-Hispanic whites were more likely to report low organizational cultural competence. Students/employees of the participating children's hospital were more likely to report higher cultural competence scores as compared with students/employees from the school of medicine (AOR: 1.92; 95% CI: 1.50–2.47). Within this site, however, there were significant differences in adjusted rates (standard error [SE]) in ranking CCF above average, with 72.0 (4.0)% males and 62.1 (2.6)% females (adjusted risk difference [SE]: 9.9 [4.8]; *p*=0.04); and 70.3 (2.5)% whites and 29.2 (7.7)% blacks (adjusted risk difference [SE]: 41.1 [8.1]; *p*<0001).

## Discussion

We analyzed health system survey data and found that respondents who self-identified as female or transgender, non-Hispanic black or multiracial/ethnic, and LGBTQ as compared with those identifying as male, non-Hispanic white, and heterosexual, respectively, reported the organization's cultural competence significantly lower. The distribution of our CCF score with a mean (SD) of 15.44 (3.0) was similar to benchmark mean (SD) of 15.46 (2.98). In addition, our Cronbach's α of 0.79 aligned with prior internal consistency measurement that revealed a Cronbach's α of 0.81 for the CCF.^10^

We expand upon the findings by Person et al.^10^ to highlight the importance of individual personal characteristics over position or length of employment. Accounting for characteristics such as gender or race/ethnicity eliminated prior differences seen by position. Respondents who had been at the organization less than a year were more likely to report higher CCF scores; however, after that first year, there were no significant differences by length of employment. Contrary to Person et al.,^10^ we found that sites with higher CCF scores did not reflect a higher percentage of minorities or females with higher CCF scores. This finding emphasizes the need for organizations to routinely stratify such metrics to effectively assess their institutional culture.

Beyond routine stratification of ongoing survey assessments of inclusion, our findings suggest that future work should explore through qualitative assessments how organizations may improve their CCF scores. Average CCF scores among organizations appear to be high with narrow SDs, akin to the high ceiling effects often seen in patient experience surveys.^14^ Future studies should center on elucidating what constitutes a meaningful change in CCF score and if and how organizations can intervene to achieve such change.

Our study had some limitations. These findings may lack generalizability, as we conducted the study in one academic healthcare system. However, our healthcare system spans three states with multiple hospitals and academic schools with the diversity of our medical student body and faculty reflective of AAMC averages (Supplementary Appendix Table A1). Our study also illustrates a generalizable approach that organizations can adopt to evaluate and intervene upon institutional culture. The survey administration method allowed for individuals present on more than one list serve to be counted more than once in our sampling population (denominator). This may have resulted in an underestimation of our response rate. Individuals completing the survey more than once or duplications in the numerator would counter this. However, we posit that individuals would be less inclined to complete multiple surveys, given the survey burden and lack of monetary incentive. The survey is voluntary and thereby subject to nonresponse bias; however, key to our analysis are perceptions from those motivated to share them. Our response rates by respondent characteristics were consistent or better than other institutions administering this survey (Supplementary Appendix Table A2).

Organizational cultural competence influences both recruitment and retention of diverse employees.^15,16^ Organizations can utilize the DES data to determine critical gaps, tailor strategic solutions, and measure potential improvements in cultural competence. These results reveal opportunities for further inquiry and improvements. Our findings led to subsequent qualitative assessments to better understand what specific CCF scores translate to in reality in effort to guide interventions.^17^ Our results suggest there is a potential lack of knowledge among executive leadership of the challenges other members of their organizations may face. Interventions should consider the importance that leaders serve in setting the tone for institutional culture and incorporate inclusive leadership skills. To that end, we have instituted the unconscious bias workshops for leadership and set system-level priorities for both measuring and achieving inclusion. Self-awareness and the role that unconscious biases may play in the assumptions we make with our colleagues are also critical to cultural transformation.^18,19^

The AAMC in collaboration with University of Massachusetts Medical School introduced the DES to allow institutions to measure their progress in creating an inclusive work and learning environments.^10,11^ Our study highlights how organizations utilize specific metrics stratified by personal characteristics for ongoing assessments and development of targeted solutions to improve institutional culture. Future work should consider stratifying DES metrics by respondent demographics to evaluate the effectiveness of interventions designed to improve inclusion as well as qualitative assessments to better understand what constitutes meaningful changes in CCF scores in efforts to guide interventions.

## Supplementary Material

Supplemental data

Supplemental data

## Abbreviations Used

AAMC: Association of American Medical Colleges
AOR: adjusted odds ratio
CCF: cultural competence factor
CIs: confidence intervals
DES: Diversity Engagement Survey
IQR: interquartile range
LGBTQ: lesbian/gay/bisexual/transgender/queer
NH: non-Hispanic
OR: odds ratio
SD: standard deviation
SE: standard error
SOM: school of medicine

## References

[1] Weiss KB, Bagian JP, Wagner R CLER pathways to excellence: expectations for an optimal clinical learning environment (executive summary). J Graduate Med Educ. 2014;6:610 10.4300/JGME-D-14-00348.1 PMC4535242 26279803

[2] Obedin-Maliver J, Goldsmith ES, Stewart L, et al. Lesbian, gay, bisexual, and transgender–related content in undergraduate medical education. JAMA. 2011;306:971–977 21900137 10.1001/jama.2011.1255

[3] Niu NN, Syed ZA, Krupat E, et al. The impact of cross-cultural interactions on medical students' preparedness to care for diverse patients. Acad Med. 2012;87:1530–1534 23018328 10.1097/ACM.0b013e31826d40f5

[4] Smith DG Building institutional capacity for diversity and inclusion in academic medicine. Acad Med. 2012;87:1511–1515 23018326 10.1097/ACM.0b013e31826d30d5

[5] Avery DR, Thomas KM Blending content and contact: the roles of diversity curriculum and campus heterogeneity in fostering diversity management competency. Acad Manag Learn Educ. 2004;3:380–396

[6] Ely RJ, Thomas DA Cultural diversity at work: the effects of diversity perspectives on work group processes and outcomes. Adm Sci Q. 2001;46:229–273

[7] Institute of Medicine. 2003 Unequal Treatment: Confronting Racial and Ethnic Disparities in Health Care. Washington, DC: The National Academies Press 10.17226/10260 25032386

[8] Cohen JJ, Gabriel BA, Terrell C The case for diversity in the health care workforce. Health Affairs. 2002;21:90–102 12224912 10.1377/hlthaff.21.5.90

[9] Shore LM, Randel AE, Chung BG, et al. Inclusion and diversity in work groups: a review and model for future research. J Manag. 2011;37:1262–1289

[10] Person SD, Jordan CG, Allison JJ, et al. Measuring diversity and inclusion in academic medicine: the diversity engagement survey. Acad Med. 2015;90:1675–1683 26466376 10.1097/ACM.0000000000000921 PMC5823241

[11] Childs G, Jones R, Nugent KE, et al. Retention of African-American students in baccalaureate nursing programs: are we doing enough? J Prof Nurs. 2004;20:129–133 15176015 10.1016/j.profnurs.2004.03.002

[12] DataStar. Interested in Using the Diversity Engagement Survey (DES)? Available at www.surveystar.com/des Accessed 72, 2018

[13] DataStar. Statement of HIPAA Compliance. Available at https://www.surveystar.com/statement%20of%20hipaa%20compliance.pdf Accessed 72, 2018

[14] Presson AP, Zhang C, Abtahi AM, et al. Psychometric properties of the Press Ganey^®^ Outpatient Medical Practice Survey. Health Qual Life Outcomes. 2017;15:32 28183312 10.1186/s12955-017-0610-3 PMC5301343

[15] McKay PF, Avery DR, Tonidandel S, et al. Racial differences in employee retention: are diversity climate perceptions the key? Pers Psychol. 2007;60:35–62

[16] Myers VL, Dreachslin JL Recruitment and retention of a diverse workforce: challenges and opportunities. J Healthcare Manag. 2007;52:290–298 17933185

[17] Aysola J, Barg FK, Martinez AB, et al. Perceptions of factors associated with inclusive work and learning environments in health care organizations: a qualitative narrative analysis. JAMA Network Open. 2018;1:e181003 10.1001/jamanetworkopen.2018.1003 PMC6324264 30646094

[18] Wuffli PA Introduction: A Framework for Inclusive Leadership. Inclusive Leadership: Springer, 2016, pp. 1–7

[19] Williams A Unconscious Bias in the Workplace—What Is It and What Role Is It Playing in the Inability of Organisations to Drive Forward on Diversity. Sydney, Australia: Johnson Partners, 2011

