# Supplementary material for: Measuring Organizational Cultural Competence to Promote Diversity in Academic Healthcare Organizations
Source: Health Equity. 2018 Nov 8;2(1):316–20. doi: 10.1089/heq.2018.0007 (PMC6231484; doi:10.1089/heq.2018.0007)
Supplement: Supplemental data [file Supp_Appendixtable1.pdf]

Supplementary Material

Supplementary Appendix Table A1. Faculty and Student Distribution by Gender and Race/Ethnicity: Our Institution Compared with Association of American Medical Colleges National Data

Characteristic	Our institution (2015)	AAMC national data (2015)
Male faculty	63.2%	60.2%
Female faculty	36.8%	39.5%
Minority faculty	26.0%	37.3%
Underrepresented minority faculty	7.1%	12.1%
Male student	55.5%	53.1%
Female student	44.5%	46.9%
URM student	21.8%	33.1%
Minority	43.8%	45.7%

AAMC, Association of American Medical Colleges; URM, underrepresented minority, which consists of Blacks, Mexican-Americans, Native Americans (that is, American Indians, Alaska Natives, and Native Hawaiians), and mainland Puerto Ricans.
