# Supplementary material for: Measuring Organizational Cultural Competence to Promote Diversity in Academic Healthcare Organizations
Source: Health Equity. 2018 Nov 8;2(1):316–20. doi: 10.1089/heq.2018.0007 (PMC6231484; doi:10.1089/heq.2018.0007)
Supplement: Supplemental data [file Supp_Appendixtable2.pdf]

Supplementary Appendix Table A2. Comparison of Our Response Rates by Characteristics with Other Institutions That Administered the Diversity Engagement Survey

	University of Pennsylvania, *n* (%)	Benchmark, *n* (%)
All respondents	3506 (100)	13,694 (100)
Gender		
Male	1197 (34)	4479 (33)
Female	2255 (64)	8811 (64)
Sexual orientation		
Heterosexual	3037 (87)	11,846 (87)
LGBTQ or other	358 (10)	950 (7)
Missing/refused to answer	111 (3)	898 (7)
Race/ethnicity		
White	2409 (69)	9789 (71)
Black/African American	379 (11)	1134 (8)
Hispanic/Latino(a)	132 (4)	738 (5)
Asian	376 (11)	1002 (7)
Other	117 (3)	487 (4)
Belief system		
Christian	1546 (44)	7811 (57)
Non-Christian	1594 (45)	4106 (30)
Length of time at school		
<1 year	550 (16)	1871 (14)
1–5 years	1313 (37)	4781 (35)
5–10 years	540 (15)	2620 (19)
≥ 10 years	1085 (31)	4180 (31)
Position		
Executive leadership	131 (4)	431 (3)
Faculty	725 (21)	1475 (11)
Staff	502 (14)	4899 (36)
Student	765 (22)	1177 (9)
Resident/fellow/housestaff	255 (7)	178 (1)
Postdoctoral	199 (6)	192 (1)
Generational age group		
Traditional (1922–1944)	74 (2)	312 (2)
Baby boomers (1945–1964)	908 (26)	5458 (40)
Generation X (1965–1980)	1018 (29)	4290 (31)
Millennials (1981–2000)	1447 (41)	3219 (24)

LGBTQ, lesbian/gay/bisexual/transgender/queer.
